# Examining the Interactions Between Expectations and tDCS Effects on Motor and Cognitive Performance

**DOI:** 10.3389/fnins.2018.00999

**Published:** 2019-01-07

**Authors:** Sheida Rabipour, Petar Sefik Vidjen, Anthony Remaud, Patrick S. R. Davidson, François Tremblay

**Affiliations:** ^1^School of Psychology, University of Ottawa, Ottawa, ON, Canada; ^2^Bruyère Research Institute, Bruyère Continuing Care, Ottawa, ON, Canada

**Keywords:** cognitive enhancement, expectation, non-invasive brain stimulation, placebo effect, transcranial direct current stimulation

## Abstract

**Background:** Despite a growing literature and commercial market, the effectiveness of transcranial direct current stimulation (tDCS) remains questionable. Notably, studies rarely examine factors such as expectations of outcomes, which may influence tDCS response through placebo-like effects. Here we sought to determine whether expectations could influence the behavioral outcomes of a tDCS intervention.

**Methods:** Through an initial study and self-replication, we recruited 121 naïve young adults 18–34 years of age (*M* = 21.14, *SD* = 3.58; 88 women). We evaluated expectations of tDCS and of motor and cognitive performance at three times: (i) at baseline; (ii) after being primed to have High or Low expectations of outcomes; and (iii) after a single session of sham-controlled anodal tDCS over the left or right motor cortex. Before and after stimulation, participants performed the Grooved Pegboard Test and a choice reaction time task as measures of motor dexterity, response time, and response inhibition.

**Results:** Repeated measures ANOVA revealed that participants had varying, largely uncertain, expectations regarding tDCS effectiveness at baseline. Expectation ratings significantly increased or decreased in response to High or Low priming, respectively, and decreased following the intervention. Response times and accuracy on motor and cognitive measures were largely unaffected by expectation or stimulation conditions. Overall, our analysis revealed no effect attributable to baseline expectations, belief of group assignment, or experimental condition on behavioral outcomes. Subjective experience did not differ based on expectation or stimulation condition.

**Conclusions:** Our results suggest no clear effects of tDCS or of expectations on our performance measures, highlighting the need for further investigations of such stimulation methods.

## 1. Introduction

Stimulating the brain non-invasively to enhance performance using methods such as transcranial direct current stimulation (tDCS) represents an enticing prospect. Relatively inexpensive and safe (Bikson et al., 2016; Matsumoto and Ugawa, 2017), tDCS is increasingly promoted for motor and cognitive enhancement in healthy and clinical populations (e.g., Coffman et al., 2014; Zhao et al., 2017). Such perceptions regarding tDCS-induced enhancement have created a growing interest in the consumer market (Brühl and Sahakian, 2016), including a burgeoning Do-It-Yourself community of non-expert tDCS enthusiasts (The Economist, 2015).

Despite a growing scientific literature, tDCS effects remain inconsistent (Bestmann et al., 2015), raising concerns over potential commercial applications in the general public (Farah, 2015; Jwa, 2015; Carter and Forte, 2016; Wurzman et al., 2016). Whereas, some studies have reported improvements in functions such as memory (Sandrini et al., 2014; Katz et al., 2017), motor and cognitive skill (Kincses et al., 2004; Hashemirad et al., 2016; Kang et al., 2016), and executive function (Dockery et al., 2009; Au et al., 2016), others suggest no reliable effect of different stimulation protocols on motor (Horvath et al., 2016; López-Higes et al., 2018), cognitive (Medina and Cason, 2017; Nilsson et al., 2017), or even basic neurophysiological (Dyke et al., 2016; Parkin et al., 2018) outcomes. Such inconsistencies have blurred interpretations of tDCS outcomes as a whole. For example, a systematic review of neurophysiological outcomes concluded that tDCS had no reliable effect beyond change in motor evoked potentials, a marker of corticospinal excitability (Hallett, 2007), outlining high variability and flawed methodology as limiting factors in comparing and pooling results across existing research (Horvath et al., 2015, 2016). These conclusions have been criticized, in turn, on the ground that the authors attempted to prematurely—and, at times, erroneously—aggregate results of tDCS studies regardless of key differences in protocol and using an inappropriate statistical approach (Antal et al., 2015; Price and Hamilton, 2015).

In addition to the variability in study protocols, researchers have raised concerns over the quality of the increasing body of work surrounding tDCS. Specifically, studies have typically included small samples, heterogeneous populations, insufficiently challenging tasks, poorly motivated participants, and failed to account for other important factors—such as individual differences in performance, biological characteristics, or psychological composure—that may influence responsiveness to tDCS interventions (Berryhill et al., 2014; Horvath et al., 2014). Examinations of technical factors such as electrode placement (Penolazzi et al., 2013; Parkin et al., 2018), intensities (Underwood, 2016; Vöröslakos et al., 2018), and stimulation schedule (Au et al., 2016) have not adequately resolved the inconsistencies in reported tDCS effects or the large intra- and inter-individual variability of response to tDCS (Wiethoff et al., 2014; Li et al., 2015; López-Alonso et al., 2015). Overcoming these limitations is imperative to determine whether tDCS protocols truly are effective and, even if genuine positive effects are possible, to identifying the boundary conditions based on individual contexts.

In particular, very few studies have examined how factors such as expectations might influence outcomes of tDCS interventions, through placebo-like effects (e.g., Boot et al., 2013; Benedetti, 2014; Schwarz et al., 2016). Incentive-based motivation may enhance the cognitive effects of tDCS (Jones et al., 2015). Expectations, moreover, have been show to modulate the effects of deep brain stimulation in clinical populations, such as patients with Parkinson's disease (Keitel et al., 2013). Mixed findings in the literature have generated polarizing claims in popular science magazines and media, which may influence expectations of outcomes at the outset of an intervention (Rabipour et al., 2017). Such expectations regarding outcomes represent a potential factor that may impact tDCS interventions aiming at modulating performance.

Previously, we found that high expectations of outcomes could enhance the effects of anodal tDCS on executive functions in young adults, whereas low expectations seemed to have opposite effects (Rabipour et al., 2018b). Here we sought to extend this finding by further examining the potential interactions between expectations of outcomes and tDCS effects on tasks that rely both on motor and cognitive function. Although reports of improvement in motor performance have been more consistent than those regarding cognitive performance (e.g., improvements in motor dexterity after single tDCS administration in healthy young Christova et al., 2015 and older adults; Parikh and Cole, 2014; Greenwood et al., 2018), the real impact of tDCS effects with respect to improvements in motor and cognitive functions remains highly controversial (Fagerlund et al., 2015). We therefore aimed to directly address such inconsistencies in the literature using a double-blind balanced-placebo design with sham control, reasonable statistical power, and commonly reported tDCS parameters.

## 2. Methods

In a series of studies, we examined the effects of tDCS and of expectation priming on tasks in which people generally have stable performance. In Study 1, participants performed a dexterity task with their preferred (dominant) hand. Study 2 was a replication and extension of Study 1 in which participants performed both a dexterity and reaction time task with their non-preferred (non-dominant) hand, to examine the potential effects of tDCS and expectation priming under conditions more amenable to performance improvement.

### 2.1. Participants

We recruited 121 healthy young adults (88 women, age = 21.1 ± 3.6 years) through the Integrated System of Participation in Research at the University of Ottawa and from the Ottawa community via ads and flyers. For our initial experiment (*Study 1*), we collected data from 58 young adults (42 women; age = 21.6 ± 3.6), with no handedness specifications. We then recruited an additional 63 right-handed young adults (46 women; age = 20.6 ± 3.5) for a follow-up replication and extension of our initial protocol (*Study 2*).

In both studies, we screened participants for health issues that could potentially interfere with performance outcomes (e.g., neurological conditions) and for contraindications to brain stimulation (e.g., history of epilepsy, presence of metal in head, pregnancy etc.) using a questionnaire (adapted from Keel et al., 2011). Participants reported handedness using a web-based version of the Edinburgh Handedness Inventory (adapted from Oldfield, 1971 see results for handedness distributions). Participants were told about possible side effects before providing consent. We received ethical approval to conduct this study from the Research Ethics Boards at the University of Ottawa and the Elizabeth Bruyère Research Institute. We registered the trial at clinicaltrials.gov (ID: NCT02498574).

### 2.2. Study Design

After providing consent, participants in each study were assigned to one of two expectation priming conditions: (i) High expectation priming, in which participants were told they would receive a type of brain stimulation known to improve performance; and (ii) Low expectation priming, in which participants were told they would receive a type of brain stimulation with no known benefits. We then randomized participants to receive one of two stimulation conditions: active anodal or sham tDCS. Random assignment of participant conditions was achieved using a numerical list representing the order of the expectation priming (high vs. low) and stimulation (active vs. sham) condition.

All participants proceeded to complete the baseline transfer measures in counterbalanced order, followed by expectation assessment and priming, the tDCS session, and, finally, the post-stimulation assessment of expectations and performance on the transfer tasks.

### 2.3. Expectation Assessment and Priming

In both Study 1 and 2, participants rated their expectations of tDCS effectiveness using the Expectation Assessment Scale (EAS), a questionnaire we described in our previous work (Rabipour and Davidson, 2015; Rabipour et al., 2017) and validated for use in this context (Rabipour et al., 2018a), on three occasions: (i) at baseline; (ii) after receiving High or Low expectation priming; and (iii) after the tDCS session. Briefly, participants rated their expectations of outcomes on a scale from 1 to 7 (Table 1). We probed expectations on nine outcomes: (i) general cognitive function; (ii) memory; (iii) concentration; (iv) distractibility; (v) reasoning ability; (vi) multitasking ability; (vii) performance in everyday activities; (viii) motor dexterity; and (ix) response time.

**Table 1:** Rating scale used in the Expectation Assessment Scale.

**Item**		
**(A) ITEMS INCLUDED IN THE QUESTIONNAIRE**		
Cognitive function.		
Memory.		
Concentration.		
Distractibility (i.e., *lowering* how much you lose focus on a task).		
Reasoning ability.		
Multi-tasking ability (i.e., managing multiple tasks at the same time).		
Performance in everyday tasks (e.g., driving, remembering important dates, managing finances, etc.)		
Motor dexterity (i.e., how *well* you manipulate objects with your fingers).		
Motor reaction time (i.e., how *fast* you respond with your fingers).		
**Rating**	**Degree of expected success**	**Definition**
**(B) RATING SCALE AND ACCOMPANYING DEFINITIONS**		
1	Completely unsuccessful	No change in brain activity or noticeable behavior. Such a procedure would be a waste of time and resources.
2	Fairly unsuccessful	Possible changes in specific brain activity (i.e., detectable at the neurological level), yet unnoticeable in daily life. Such a procedure would be a waste of time and resources.
3	Somewhat unsuccessful	Possible changes in general brain activity (i.e., detectable at the neurological level), yet unnoticeable in daily life.
4	I have absolutely no expectations	*Neutral rating; additional definition not provided*.
5	Somewhat successful	Possible changes in specific brain activity and behavior. Such a procedure would NOT be a waste of time or resources.
6	Fairly successful	Possible changes in general brain activity as well as noticeable behavioral changes.
7	Completely successful	Changes in general brain activity as well as noticeable changes in overall thought and behavior that positively impact daily life. Such a procedure would be a good investment of time and resources.

*Participants rated the degree to which they predicted the stimulation would be successful (before stimulation) or felt the stimulation was successful (after stimulation) at improving each ability. Definitions and formatting shown as provided in the survey*.

*Participants were shown the definition only for the first item; for efficiency, subsequent items displayed only the options for degree of expected success*.

Experimenters were not blinded to the expectation priming condition as this knowledge was necessary for them to administer the appropriate version of the questionnaire. Participants were not informed about the different expectation priming conditions in the study and only read one of the two priming messages, based on their randomly assigned condition. The experimenters told participants that the survey contained important information about the study, without providing additional details.

### 2.4. Study 1: Stimulation Over Primary Motor Cortex Representing the Preferred Hand

#### 2.4.1. Stimulation Procedure

To determine the location of the hand representation in the primary motor cortex (M1) in each participant, single pulse transcranial magnetic stimulation (TMS) was delivered via a Magstim 200 stimulator (MagStim Corp., Dyfed U.K.) connected to a figure-eight coil (70 mm inner loop). TMS-induced motor evoked potentials (MEPs) were recorded in hand muscles using small surface electrodes (Delsys 2.1, Bagnoli EMG, Delsys, Inc) placed over the first dorsal interosseus (FDI) muscle. Using a suprathreshold intensity (1.1 x motor threshold), the area over the scalp was explored with the coil in 1cm steps until reliable MEPs (>50 microVolts) could be elicited in FDI. We then marked this site with a felt pen on the scalp. To deliver the stimulation current, we used a programmable battery-driven direct current (DC) stimulator (HDCStim, Newronika, Milano, Italy) coupled with a pair of silicone-rubber electrodes encased in 5 x 7 cm sponges soaked in 0.9% saline. The montage corresponded to the “classical motor learning montage” (Nitsche et al., 2008) with the anode placed over the M1 region (i.e., area representing the motor cortex as defined with TMS) and the cathode located on the supraorbital region on the opposite side. The electrodes were held against the head with elastic bandages. These parameters are in-line with those reported to yield relatively consistent outcomes (Ho et al., 2016).

For the tDCS intervention, the stimulator was pre-programmed by investigators (AR and FT) who did not intervene in the tDCS application, to deliver a constant current of 2.0 mA (density of 0.054 mA/cm^2^) for a duration of 20 min with a ramp-up and ramp-down of 30 s. In the active condition, the DC stimulation was maintained for the whole duration. For the sham condition, we delivered current only during the ramp-up and ramp-down phases; the current was held at 0.0 mA for the remainder of the protocol. This allowed participants in each group to experience the same initial sensations (i.e., mild tingling). These stimulation parameters have been previously validated for safety and potential cognitive effects in healthy participants (Iyer et al., 2005; Bikson et al., 2016), as well as for consistency in motor outcomes (Ammann et al., 2017; Jamil et al., 2017). Both participants and experimenters were blind to the stimulation condition.

#### 2.4.2. Stimulation Task

During the 20-minute tDCS application (active or sham), participants performed the *Finger Fitness* task (Motrix ©available via iTunes), a game-based reaction time (RT) task, on a touch-screen electronic tablet (iPad). The task involved tapping fingers as fast as possible on the tablet according to a pre-defined sequence, indicated by a change in color of the target location. We used this task as a means to ensure that participants remained vigilant and focused on their dexterity performance during the stimulation period, rather than an outcome measure *per se*.

#### 2.4.3. Outcome Measures

*Grooved Pegboard Test (GPT)*: The GPT is a standardized, commonly used test requiring participants to place pegs (*n* = 25) into the grooves of a board placed directly in front of them, as quickly as possible. The test requires complex visuo-sensori-motor integration to rotate pegs to the correct orientation before placing them in the hole. Studies have demonstrated the reliability and degree of challenge of the GPT across the lifespan (Wang et al., 2011). Although primarily used as an index of manual dexterity, the GPT can also capture cognitive function because its performance relies on executive control (e.g., decision making, monitoring of performance) (Ashendorf et al., 2009; Bezdicek et al., 2014; Vasylenko et al., 2018). Moreover, tDCS applied during training may improve GPT performance in young adults (Christova et al., 2015), although the evidence for this effect remains sparse and inconsistent (Fagerlund et al., 2015). Given the ability of the GPT to reflect not only motor performance but also cognitive processing, we elected to use this index as an outcome in our tDCS intervention. We allotted participants one practice trial at baseline and then assessed their performance (i.e., time to complete the board) on a test trial with both the preferred and non-preferred hand, before and after the stimulation procedure.

#### 2.4.4. Feedback on Perceived Experience

Following the stimulation procedure, we asked participants to complete a satisfaction questionnaire for feedback regarding their perceived experience. On a scale of 1–7 (Table 2), participants rated the degree to which they found the experience: (i) enjoyable; (ii) challenging; (iii) frustrating; (iv) engaging; (v) boring; (vi) motivating; and (vii) satisfying.

**Table 2:** Questions and rating scales included in the subjective feedback questionnaire.

**Item**		
**(A) FEEDBACK QUESTIONS (i.e., ITEMS)**		
I found the program to be enjoyable.		
I found the program to be challenging.		
I found the program to be frustrating.		
I found the program to be engaging.		
I found the program to be boring.		
I was motivated to do the brain stimulation program.		
How satisfied were you with this program?		
**Rating**	**Agreement scale**	**Satisfaction scale**
**(B) RATING SCALES**		
1	Very strongly disagree	Extremely dissatisfied
2	Strongly disagree	Fairly dissatisfied
3	Disagree	Somewhat dissatisfied
4	Neither agree nor disagree	Neither satisfied nor dissatisfied
5	Agree	Somewhat satisfied
6	Strongly agree	Fairly satisfied
7	Very strongly agree	Extremely satisfied

*Participants rated the extent to which they agreed with each statement, and finally the degree to which they were satisfied with the overall experience*.

### 2.5. Study 2: Stimulation Over Primary Motor Cortex Representing the Non-preferred Hand

#### 2.5.1. Stimulation Procedure

In a separate sample of participants, we used the same TMS protocol to locate the hand motor area (M1) representing the non-preferred hand. The tDCS protocol was also similar, using the same montage as Study 1, except that the anodal stimulation was directed at the M1 of the non-preferred hand. The stimulation conditions and task were identical to those in Study 1.

#### 2.5.2. Outcome Measures

*GPT*: In Study 2, participants were also tested on the GPT, but only with the non-preferred hand. The testing protocol with the GPT was otherwise identical.

*Additional outcomes*: In addition to the GPT, we assessed participants on: (1) the Finger Tapping test and (2) the Choice Reaction Time (CRT) test, both performed with the non-preferred hand. We administered the two tests using a MoART panel (Lafayette Instruments) with the accompanying software (PsymSoft II™). For the finger tapping test, participants were asked to repeatedly tap their index finger on a target location on the board as quickly as possible in response to a “go” cue (green light), for the duration of the cue (30 s). For the CRT test, participants were asked to release their index finger from the target location on the board in response to the “go” cue (green light), but not in response to one of two possible “no-go” cues: (i) red light; or (ii) green light paired with a tone. RT on the CRT test were measured in milliseconds from stimulus onset to response, averaged over five blocks of 20 trials.

#### 2.5.3. Feedback on Perceived Experience

In addition to the satisfaction questionnaire administered in Study 1, we probed participants' expectations and experience of side effects commonly reported in tDCS interventions (Brunoni et al., 2011; Kessler et al., 2012), including itchiness, warmth/heat, pinching, pain, iron taste, fatigue, or other.

### 2.6. Statistical Analysis

We performed analyses using IBM SPSS Statistics, Inc. version 24, R version 3.1.1, and JASP version 0.9.1 (JASP Team, 2018). For both experiments, we determined sample sizes a priori using G^*^Power version 3.1, based on a moderate effect size (Cohen's f(V) = 0.45), alpha level of 0.05, and 80% power.

In both studies we analyzed primary outcomes using repeated measures analysis of variance (ANOVA), as well as univariate ANOVA and multivariate ANOVA (MANOVA), with within-between factors interactions to evaluate the outcome measures described, at an alpha level of 0.05. Where applicable, we used the Holm-Bonferroni correction for multiple comparisons and Greenhouse-Geisser correction for sphericity. We followed up our analysis of performance outcomes with Bayesian repeated measures ANOVA, using the default parameters in JASP.

## 3. Results

We excluded from our analyses four women and two men who did not complete the study protocol in Study 1 due to hair thickness preventing proper application of tDCS (*n* = 5) and anxiety regarding the protocol (*n* = 1). We have made our data available on Open Science Framework (https://osf.io/54k7a/).

Participants in both studies were matched across experimental conditions, with no significant group differences in age, sex, prior knowledge of NIBS methods, prior experience with NIBS, concern over declining cognitive function, or use of medications for emotional or mood-related issues within the past five years (Table 3). These proportions did not significantly differ based on stimulation site (i.e., between our initial and follow-up experiments).

**Table 3:** Sample composition in Study 1 and 2.

**Group**	**Stimulation**	**Expectation priming**	***n* (women)**	**Age *(SD)***	**Prior knowledge**	**Prior experience**	**Concern**	**Medication use**
**(A) STUDY 1 PARTICIPANT DEMOGRAPHICS**								
1	Active	High	13 (9)	22.15 *(3.53)*	75%	23%	15%	33%
2	Active	Low	12 (10)	21.75 *(3.60)*	75%	15%	33%	25%
3	Sham	High	13 (11)	20.62 *(2.40)*	75%	23%	38%	27%
4	Sham	Low	14 (8)	22.00 *(4.62)*	54%	7%	21%	15%
**(B) STUDY 2 PARTICIPANT DEMOGRAPHICS**								
1	Active	High	18 (12)	20.44 *(3.79)*	67%	22%	19%	17%
2	Active	Low	14 (9)	21.00 *(3.68)*	57%	14%	23%	35%
3	Sham	High	18 (14)	19.78 *(2.51)*	56%	44%	42%	28%
4	Sham	Low	13 (11)	21.38 *(4.07)*	69%	15%	38%	46%

*Groups were balanced with respect to distribution of sex, mean age, prior knowledge of and experience with NIBS, concern over declining cognitive function, and use of medications for emotional or mood-related issues*.

*Notably, although the majority of participants had at least some prior knowledge of NIBS, few had any experience with such techniques*.

The handedness questionnaire revealed four left-handed women and one ambidextrous woman in Study 1. Results did not differ qualitatively on the basis of handedness; we therefore elected to retain these participants in our analyses.

### 3.1. Expectation Effects

Patterns of expectation ratings did not significantly differ between Study 1 and 2; we therefore combined data from the two studies in our analyses of these results.

Participants were largely optimistic of NIBS outcomes at baseline: ratings in most domains were significantly above neutral [*t*_(114)_ ≥ 3.3, *p* ≤ 0.001, Cohen's *d* ≥ 0.63], with the exception of “reasoning” [*t*_(114)_ = 1.93, *p* = 0.056], “multitasking” [*t*_(114)_ = 0.27, *p* = 0.787], and “performance in everyday activities” [*t*_(114)_ = 1.08, *p* = 0.283]. Mean expectation ratings did not significantly differ across groups at baseline. Because of missing responses (*n* = 16) in the “motor dexterity” and “response time” domains, we dropped those from further analyses.

**Figure 1:**
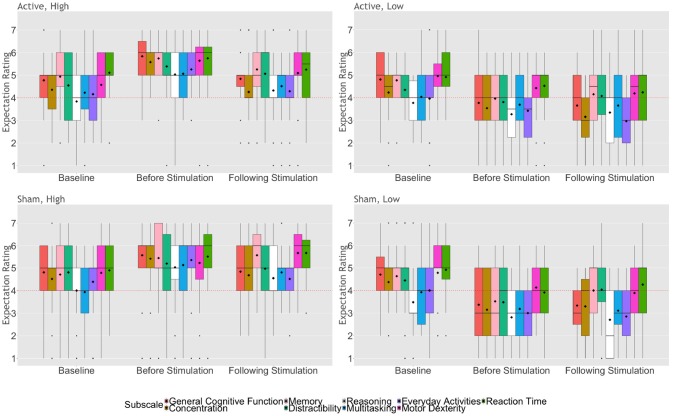
Expectation ratings of NIBS outcomes across time, based on experimental condition, collapsed across Study 1 and 2. The upper and lower whiskers represent 1.5 x the inter-quartile range. Bold horizontal lines represent group medians; diamonds represent group means. Dots represent outliers. Dashed lines indicate a neutral score (rating of “4”).

Repeated measures MANOVA comparing expectation ratings across the remaining seven domains between stimulation and expectation condition at baseline, after receiving the expectation priming message, and after stimulation revealed a significant main effect of time [Wilk's λ = 0.736, *F*_(14, 432)_ = 5.10, *p* < 0.0001, $\eta_{p}^{2}$ = 0.14] and of expectation condition [Wilk's λ = 0.669, *F*_(14, 105)_ = 7.42, *p* < 0.0001, $\eta_{p}^{2}$ = 0.33], as well as a significant interaction between time and expectation condition [Wilk's λ = 0.648, *F*_(14, 432)_ = 7.49, *p* < 0.0001, $\eta_{p}^{2}$ = 0.20; Figure 1]. We did not find a main effect of stimulation condition on expectation ratings in any of the domains.

Further examination of the ratings for each cognitive domain demonstrated a significant effect of time for general cognitive function [*F*_(1.8, 199.7)_ = 11.52, *p* < 0.0001, $\eta_{p}^{2}$ = 0.09], memory [*F*_(2, 222)_ = 12.85, *p* < 0.0001, $\eta_{p}^{2}$ = 0.10], and performance in everyday activities [*F*_(1.9, 209.7)_ = 12.22, *p* < 0.0001, $\eta_{p}^{2}$ = 0.10], as well as a significant effect of expectation condition [*F*_(1, 111)_ ≥ 23.68, *p* < 0.0001, $\eta_{p}^{2}$ = 0.18] and a significant interaction between time and expectation condition [*F*_(1.7, 338.2)_ ≥ 8.76, *p* ≤ 0.001, $\eta_{p}^{2}$ = 0.07] for all domains. Specifically, after reading their respective expectation messages, participants who received high expectation priming rated higher expectations of outcomes on all domains [*t*_(61)_ ≥ 3.49, *p* ≤ 0.001, Cohen's *d* ≥ 0.89], whereas those in the low expectation conditions rated lower expectations on all domains [*t*_(52)_ ≥ 2.92, *p* ≤ 0.005, Cohen's *d* ≥ 0.81], compared to baseline. Following stimulation, participants assigned to the high expectation condition rated significantly lower expectations of improvement in general cognitive function [*t*_(61)_ = 5.23, *p* < 0.0001, Cohen's *d* = 1.34], memory [*t*_(61)_ = 6.69, *p* < 0.0001, Cohen's *d* = 1.71], reasoning ability [*t*_(61)_ = 3.94, *p* < 0.0001, Cohen's *d* = 1.00], and performance in everyday activities [*t*_(61)_ = 5.31, *p* < 0.0001, Cohen's *d* = 1.36]. Conversely, ratings of participants in the low expectation priming condition did not significantly differ following stimulation, compared to their ratings after first receiving the expectation priming message. Finally, we found that participants who received high expectation priming maintained higher expectations of outcomes following stimulation, compared to those who received low expectation priming [*t*_(113)_ ≥ 3.30, *p* ≤ 0.001, Cohen's *d* ≥ 0.62].

We also probed whether participants believed the priming they received was convincing enough to change their initial expectations, as well as the extent to which participants thought the priming messages were persuasive before and after the stimulation protocol. We found that participants who received high expectation priming were more likely to be convinced by the priming message (45/62 = 73%), compared to those who were primed to have low expectation (26/53 = 49%; *X*^2^ = 6.69, *p* = 0.01). These ratings did not differ based on stimulated hemisphere.

Repeated measures ANOVA examining participant reports of persuasiveness based on experimental condition and stimulated hemisphere showed a main effect of time [*F*_(1, 111)_ = 13.86, *p* < 0.0001, $\eta_{p}^{2}$ = 0.11], of stimulation [*F*_(1, 107)_ = 6.11, *p* = 0.015, $\eta_{p}^{2}$ = 0.05], and of expectation condition [*F*_(1, 107)_ = 13.90, *p* < 0.0001, $\eta_{p}^{2}$ = 0.12], as well as an interaction between stimulation condition and stimulated hemisphere [*F*_(1, 107)_ = 11.94, *p* = 0.001, $\eta_{p}^{2}$ = 0.10]. Specifically, participants who received active anodal stimulation to the preferred hemisphere reported being significantly less convinced by the priming message following stimulation, compared to participants who received sham stimulation to the preferred hemisphere [*F*_(1, 50)_ = 11.46, *p* = 0.001, $\eta_{p}^{2}$ = 0.19]). Moreover, participants assigned to receive high expectation priming were significantly less convinced by the priming message following stimulation, compared to those who received low expectation priming [*t*_(61)_ = 4.11, *p* < 0.0001, Cohen's *d* = 1.05].

### 3.2. Performance Effects

#### 3.2.1. Study 1: Stimulation Over Primary Motor Cortex Representing the Preferred Hand

**Figure 2:**
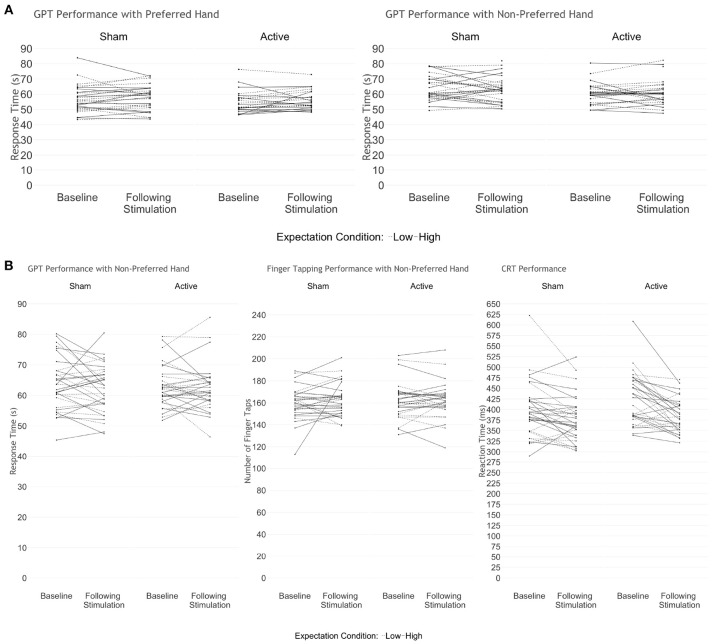
Performance on the motor transfer tasks before and after stimulation in **(A)** Study 1 and **(B)** Study 2. We found no significant differences based on stimulation or expectation condition on the transfer measures in either study. Solid lines represent participants who received high expectation priming; dashed lines indicate participants who received low expectation priming.

We performed traditional and Bayesian repeated measures ANOVA examining GPT performance with the preferred and non-preferred hand, before and after stimulation. We found no significant differences on the basis of time [baseline vs. following stimulation; *F*_(2, 47)_ = 2.14, *p* = 0.13, BF_10_ = 0.162], stimulation [active vs. sham; *F*_(2, 47)_ = 0.577, *p* = 0.57, BF_10_ = 0.507] or expectation priming condition [high vs. low; *F*_(2, 47)_ = 0.332, *p* = 0.72, BF_10_ = 0.368], and no interaction effect (BF_10_ = 0.009; Figure 2A).

#### 3.2.2. Study 2: Stimulation Over Primary Motor Cortex Representing the Non-preferred Hand

Repeated measures MANOVA examining GPT and finger-tapping performance with the non-preferred hand before and after stimulation revealed no difference in either outcome on the basis of time [*F*_(2, 58)_ = 1.107, *p* = 0.34, BF_10_ = 0.146], stimulation [*F*_(2, 58)_ = 0.117, *p* = 0.89, BF_10_ = 0.159], or expectation condition [*F*_(2, 58)_ = 0.09, *p* = 0.91, BF_10_ = 0.159], and no interaction effect (BF_10_ = 0.002; Figure 2B). Examining CRT accuracy and RT revealed no group differences at baseline. Because of a ceiling effect causing little variance in hit rate, we examined only RT in further analyses. Repeated measures MANOVA examining RT over the five trials between groups, across time revealed a significant effect of time [Wilk's λ = 0.709, *F*_(1, 59)_ = 24.24, *p* < 0.0001, $\eta_{p}^{2}$ = 0.29,BF_10_ = 1.55^*^10^12^], wherein all participants responded faster in all trials following stimulation, compared to baseline [*t*_(62)_ = 4.96, *p* < 0.0001, Cohen's *d* = 1.26]. However, we found no significant effect of trial (BF_10_ = 0.003), expectation (BF_10_ = 0.273), or stimulation condition (BF_10_ = 0.477), and no interaction between any of the variables on RT (BF_10_ ≤ 0.353).

### 3.3. Subjective Experience

#### 3.3.1. Overall Experience

Participants' feedback on their overall experience did not significantly differ between our two studies. We therefore combined analyses of these ratings across both Study 1 and 2.

**Figure 3:**
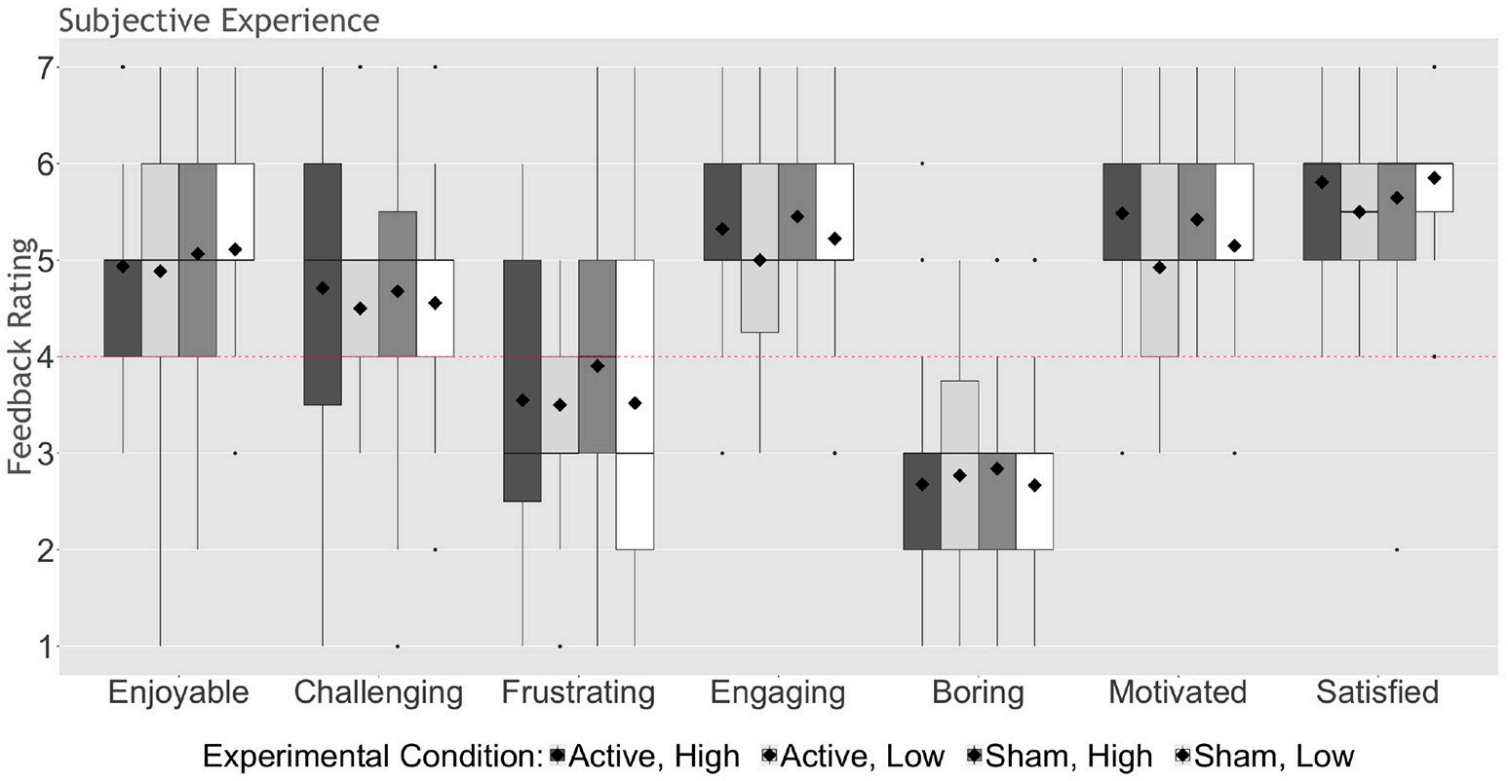
Participant feedback on experience, collapsed across Study 1 and 2. On a scale of “1” (lowest) to “7” (highest), participants rated the degree to which they found the program to be enjoyable, challenging, frustrating, engaging, boring, motivating, and satisfying (see Table 2). The upper and lower whiskers represent 1.5 x the inter-quartile range. Bold horizontal lines represent group medians; diamonds represent group means. Dots represent outliers. Dashed lines indicate a neutral score (rating of “4”).

The majority of participants in all groups (≥81%) reported believing they received active stimulation; these proportions did not significantly differ based on experimental group (*X*^2^ = 2.52, *ns*). Moreover, participants largely reported having a positive experience, with ratings of enjoyment [*t*_(25)_ ≥ 3.17, *p* ≤ 0.004, Cohen's *d* ≥ 1.27], engagement [*t*_(25)_ ≥ 5.20, *p* < 0.0001, Cohen's *d* ≥ 2.08], motivation [*t*_(25)_ ≥ 4.17, *p* < 0.0001, Cohen's *d* ≥ 1.67], and satisfaction [*t*_(25)_ ≥ 6.93, *p* < 0.0001, Cohen's *d* ≥ 2.77], significantly above neutral, and ratings of boredom [*t*_(25)_ ≥ 4.60, *p* < 0.0001, Cohen's *d* ≥ 1.84] significantly below neutral, in all groups (Figure 3). Interestingly, after correcting for multiple comparisons, only participants assigned to the high expectation priming conditions reported feeling challenged by the stimulation task [*t*_(29)_ ≥ 2.53, *p* ≤ 0.017, Cohen's *d* ≥ 0.94].

#### 3.3.2. Study 2: Experience of Side Effects

Although participants in both studies were notified of possible side effects at the outset of the experiment, we examined reports of anticipated and experienced side effects in our final 40 participants in Study 2. Before stimulation, the majority of participants in all groups (≥58%) reported having no expectation of experiencing any side effects (*X*^2^ = 2.41, *ns*). Nevertheless, following stimulation, the majority of participants in all groups (≥75%) reported experiencing at least one side effect. Chi-squared analyses revealed no significant difference in the proportion of any of the reported side effects between groups (Table 4), or based on belief of stimulation received.

**Table 4:** Proportion of participants who reported experiencing side effects following stimulation.

**Group**	**Stimulation**	**Expectation priming**	**Itchiness**	**Warmth / heat**	**Pinching**	**Pain**	**Iron taste**	**Fatigue**
1	Active	High	1/8 (13%)	4/8 (50%)	2/8 (25%)	0/8 (0%)	0/8 (0%)	1/8 (13%)
2	Active	Low	8/11 (73%)	7/11 (64%)	2/11 (18%)	1/11 (9%)	1/11 (9%)	2/11 (18%)
3	Sham	High	5/13 (38%)	10/13 (77%)	6/13 (46%)	1/13 (8%)	2/13 (15%)	0/13 (0%)
4	Sham	Low	3/8 (38%)	2/8 (25%)	3/8 (38%)	0/8 (0%)	0/8 (0%)	1/8 (13%)

*Reports of side effects did not differ based on stimulation or expectation condition; data missing from 23 participants who did not receive these questions during the experiment*.

### 3.4. Exploring Individual Factors

We explored whether any factors related to participant background (i.e., baseline expectations, sex, prior knowledge of or experience with brain stimulation, media exposure, programming experience, gaming, medication use, and concern over declining cognitive function) or performance might influence baseline expectations or performance outcomes. We found a significant effect of baseline performance on the GPT with the non-preferred hand in Study 1 [*t*_(37.05)_ = 2.97, *p* = 0.003, Cohen's *d* = 0.98], and the CRT (RT) in Study 2 [*t*_(61)_ = 4.91, *p* < 0.0001, Cohen's *d* = 1.26]. After correcting for multiple comparisons, we found no significant effect of baseline expectations, sex, prior knowledge of or experience with brain stimulation, media exposure, programming experience, gaming, medication use, or concern over declining cognitive function on performance changes in either Study 1 or 2.

Based on findings from previous research (Wong et al., 2018), we analyzed changes in performance outcomes based on the time of day in which the testing session occurred. Repeated measures MANOVA revealed no effect of session time (i.e., morning vs. afternoon) on GPT performance in Study 1 [*F*_(2, 49)_ = 0.407, *p* = 0.67], and only a marginal effect on performance outcomes in Study 2 [*F*_(7, 55)_ = 2.092, *p* = 0.06]. We found no interaction between time (i.e., baseline vs. following stimulation) and session time in either Study 1 [*F*_(2, 49)_ = 0.449, *p* = 0.64] or Study 2 [*F*_(7, 55)_ = 1.544, *p* = 0.17].

## 4. Discussion

Here we sought to examine possible interactions between expectations of tDCS outcomes and actual effects of a tDCS intervention in the context of a double-blind, sham-controlled study. Our balanced-placebo design involved stimulation conditions shown to have relatively good consistency in reported effects [i.e., current intensity (Jamil et al., 2017), electrode size and montage (Ho et al., 2016)], as well as comparable outcome measures.

### 4.1. Expectations of Outcomes

As previously reported (Rabipour et al., 2017, 2018b), we found that participant expectations of outcomes were variable at the outset, with mean ratings suggesting relatively neutral or mildly optimistic expectations of improvement on the probed domains. Also as expected, we found that our expectation priming manipulation was effective: participants who were primed to have high expectations of outcomes significantly increased their expectation ratings compared to baseline, whereas those who received low expectation priming significantly decreased their ratings. However, following the stimulation procedure, we found that participants who were primed to have high expectations significantly decreased their ratings on a number of the probed domains, whereas participants who were primed to have low expectations maintained similar ratings. Perhaps unsurprisingly, these ratings suggest a stronger effect of personal experience over our expectation priming manipulation.

Interestingly, participants who received high expectation priming maintained higher expectations of outcomes compared to those who received low expectation priming, regardless of stimulation condition. Similarly, participants who received high expectation priming were more likely to report being convinced by the information they received. These findings suggest that positive information about intervention outcomes may me more readily accepted and that receiving positive information may help raise subsequent expectations of outcomes and credulity, at least in self-reports by non-experts.

In contrast, we found that receiving active stimulation to the hemisphere representing the preferred hand (i.e., in Study 1) or high expectation priming correlated with lower self-reported persuasiveness of the priming messages. Taken together with the aforementioned findings on expectation ratings, particularly following stimulation, our results suggest that participants might not have been aware of the malleability of their expectation ratings.

### 4.2. Performance Outcomes

We found no effect of expectation or stimulation condition on any of our performance outcomes in either Study 1 or 2, including the GPT (preferred and non-preferred hand), finger tapping, or CRT performance. On the one hand, our failure to detect changes in performance based on experimental condition supports recent studies challenging the potential of tDCS to enhance motor learning (Horvath et al., 2016). On the other hand, although we selected tasks (GPT and CRT) commonly used in studies focusing on motor performance, our outcomes might not have been sensitive enough to detect changes in performance resulting from the intervention. This seems particularly true of CRT hit rate, where the absence of an effect of time and low variance suggest a possible ceiling effect. In this regard, it is possible that more challenging tasks involving learning of new motor sequences may have been more suitable to detect tDCS effects, as well as more susceptible to priming (de Xivry and Shadmehr, 2014; Savic and Meier, 2016). Along the same line, the fact that our GPT performance measure was limited to overall time may have reduced our ability to detect more subtle changes affecting sub-task components, such as time for selection, transport, insertion, and removal—all of which appear particularly sensitive to interference effects (Bryden and Roy, 2005; Almuklass et al., 2017, 2018).

### 4.3. Subjective Experience Following tDCS Protocol

Feedback reports suggest that participants had a positive experience, and were engaged with the tasks as well as motivated to perform well, regardless of experimental condition. Perhaps most importantly, we found that the majority of participants believed they received active stimulation, confirming the effectiveness of our blinding. Similarly, we found that most participants experienced at least one side effect, regardless of experimental condition or belief that they received active stimulation. Interestingly, as in our previous study of tDCS and expectations (Rabipour et al., 2018b), we found that participants assigned to the active stimulation and low expectation priming condition reported experiencing the highest proportion of itchiness; however, the reports suggest that those who received sham stimulation and high expectation priming experienced the greatest proportion of side effects overall. Although this may indicate a possible influence of expectations over perceived sensations during stimulation, these results were not statistically significant in our sample and should be further evaluated in future studies. Together, these findings suggest that our protocol was tolerable and believable, and that our performance outcomes are unlikely to be explained by differences in subjective experience. Moreover, despite recent reports showing lack of appropriate blinding to sham tDCS protocols (Greinacher et al., 2018; Turi et al., 2018), the majority of our participants reported believing they had received active stimulation and the proportion did not significantly differ between groups, suggesting that our blinding was effective.

### 4.4. Limitations and Future Considerations

Although we found no effects of either stimulation or expectation priming in our protocol, studies suggest that repeated applications of tDCS over multiple sessions could yield more consistent results both at the motor and cognitive levels (e.g., improved working memory) (Berryhill, 2017). Multi-session interventions further enable the possibility to assess learning, which may interact differently with tDCS and expectancy effects. Whether repeated tDCS applications over long-term interventions may induce larger effects and be more prone to expectations manipulations (e.g., through indirect factors such as participant motivation and engagement in the intervention; Boot et al., 2013) remains an open questions for futures studies. Similarly, the effects of tDCS administration may have been more pronounced after longer delay (Fujiyama et al., 2014); nevertheless, the majority of studies suggests greatest effects immediately following application of tDCS (Christova et al., 2015; Rumpf et al., 2017). Moreover, although we employed a conventional tDCS montage using two electrodes encased in 35 cm^2^ sponges, different electrode configurations (e.g., high-density montages) may have yielded more potent outcomes (Pixa et al., 2017). Finally, as we discuss above, it is possible that our outcome measures were not sensitive enough to detect subtle changes in performance resulting from our intervention.

## 5. Conclusion

Collectively, the present results indicate that a single session of anodal tDCS had no clear effects over sham stimulation on outcome measures reflecting motor dexterity/cognition and response time. Our results further show differences in expectations of tDCS at the outset, but suggest that priming participants' expectations of tDCS did not influence the measured outcomes. Our findings align with the current debate questioning the efficacy of tDCS as a performance enhancer and highlight the need for further investigations of such stimulation methods as well as factors (e.g., related to expectancy) which may influence results.

## Author Contributions

SR, FT and PD designed the study. SR and PV collected the data with support from AR and FT. SR, PD, and FT analyzed the data. SR wrote and revised the manuscript based on edits from all co-authors.

### Conflict of Interest Statement

The authors declare that the research was conducted in the absence of any commercial or financial relationships that could be construed as a potential conflict of interest.
